# The imported infections among foreign travelers in China: an observational study

**DOI:** 10.1186/s12992-022-00893-7

**Published:** 2022-11-24

**Authors:** Qiang Xu, Zhi-Wei Li, Xiao-Ai Zhang, Meng-Yang Liu, Jin-Long Wang, Hai-Yang Zhang, Li-Ping Wang, Xiu-Hua Guo, Li-Qun Fang, Wei Liu

**Affiliations:** 1 State Key Laboratory of Pathogen and Biosecurity, Beijing Institute of Microbiology and Epidemiology, 20 Dong-Da Street, Fengtai District, Beijing, 100071 People’s Republic of China; 2 School of Public Health, Capital Medical University, 10 Xi-Tou-Tiao, You-An-Men Street, Fengtai District, Beijing, 100069 People’s Republic of China; 3 Beijing Municipal Key Laboratory of Clinical Epidemiology, Capital Medical University, Beijing, People’s Republic of China; 4 Division of Science and Technology, Chinese Center for Disease Control and Prevention, Beijing, People’s Republic of China; 5 School of Public Health, Anhui Medical University, Hefei, People’s Republic of China

**Keywords:** Globalization, Imported infectious diseases, Public health impact, Epidemiological characteristics, China

## Abstract

**Background:**

In the past few decades, globalization has rendered more frequent and intensive population movement between countries, which has changed the original disease spectrum and brought a huge health impact on the global population including China. This study aims to describe the spectrum and epidemiological characteristics of imported infections among foreign travelers travelling to China.

**Methods:**

The data on imported infections among foreign travelers were obtained from Custom Inbound Screening System (CISS) and the National Notifiable Infectious Disease Reporting System (NNIDRS). All the infections were classified into respiratory, gastrointestinal, vector-borne, blood/sex-transmitted and mucocutaneous diseases, of which case numbers and incidences were calculated and the proportions were compared among subgroups.

**Results:**

In total, 17,189 travelers diagnosed with 58 imported infectious diseases were reported from 2014 to 2018, with an overall incidence of 122.59 per million. Respiratory infection (7,351 cases, mainly influenza) and blood/sex-transmitted diseases (6,114 cases mainly Hepatitis B and HIV infection) were the most frequently diagnosed diseases, followed by vector-borne infections (3,128 cases, mainly dengue fever and malaria). The highest case number was from Asia and Europe, while the highest incidence rate was from Africa (296.00 per million). When specific diagnosis was compared, both the highest absolute case number and incidence were observed for influenza. An obvious seasonal pattern was observed for vector-borne diseases, with the annual epidemic spanning from July to November. The origin–destination matrices disclosed the movement of imported infection followed specific routes.

**Conclusions:**

Our study provided a profile of infectious diseases among foreign travelers travelling to China and pinpointed the target regions, seasons and populations for prevention and control, to attain an informed control of imported infections in China.

**Supplementary Information:**

The online version contains supplementary material available at 10.1186/s12992-022-00893-7.

## Introduction

Before the COVID-19 epidemic, international travel had persistently increased in frequency driven by globalization, with the imported infectious diseases causing an increasingly serious public health concern faced by almost all countries. Foreign travelers or immigrants account for a high proportion of all travelers, with a critical risk of spreading the diseases across borders by which pathogens can reach new human populations or animal communities. Particularly for the vector-borne disease, the movement of patients beyond endemic countries has threatened long-term eradication goals, such as malaria [1]. Moreover, the imported cases continue to pose challenges for diagnosis and management for physicians in non-endemic areas, where it can be difficult to treat and result in high mortality. Health care providers need a complete understanding of the spectrum of imported infectious diseases and their epidemic patterns, which is critical in containing the potential epidemic or pandemic caused by imported diseases.

In China, the number of foreign travelers has steadily increased from 83.44 million person-times in 2000 to 141.20 million person-times in 2018, associated with increased reports of imported infections from 5,261 cases in 2014 to 10,157 in 2016 and 14,700 in 2018 [2, 3]. The imported infection was mainly captured by the costume entrance quarantine and national surveillance system on travel related disease [2–4], which had provided valuable information about the composition, and trends of imported infections. However, infection spectrum and the time when travel-related infection was present among foreign travelers were significantly different with other arriving travelers [2, 3], and the specific infection spectrum and epidemiological characteristics of them remained unclear, which might impede their timely and accurate diagnosis, treatment and management, especially those infections that are not endemic or rare in China. In the current study, we focused on infection diagnosed from the foreign travelers (here referred to as those with foreign citizenship) to describe the pathogen spectrum and their related epidemiological features, and explore their potential impacts on public health.

## Material and methods

### Data collection

Full details of obtaining the data on international imported infections had been provided in a previous study [4]. Briefly, data were extracted from two anonymous and delinked databases**:** the Custom Inbound Screening System (CISS) that had covered all 272 entry-exit ports in China (Figure S1), and the National Notifiable Infectious Disease Reporting System (NNIDRS) of Chinese Center for Diseases Control and Prevention (CDC) which covered all 31 provinces in the mainland of China. The data extraction process and the criteria of inclusion and exclusion were shown in Figure S2. For diagnosis of a specific disease, SOP released by the General Administration of Quality Supervision, Inspection and Quarantine of the People’s Republic of China (AQSIQ) was followed that covered all processes of sample collection and laboratory tests (Figure S3) [2]. For the screened patients, a standard questionnaire interview was applied to record the clinical and epidemiological data that were related to the travel illness (Figure S4). A predefined standard dataset was used to extract the data from the patient’s medical records, including the travel related epidemiological information, and clinical data that supported the diagnosis. For the purpose of calculating the incidence rate, the inbound arrivals data in the mainland of China were obtained from the Annual Report of China Tourism Statistics (ARCTS) 2015 to 2019. The yearly and monthly numbers of foreign travelers from 2014 to 2018 were calculated for six continents and 23 selected countries with available data about foreign travelers in the ARCTS.

### Definitions and classifications of imported infections

Altogether 67 infectious diseases were screened from two reporting systems, based on which five syndromic diseases were classified, including respiratory, gastrointestinal, vector-borne disease, blood/sex-transmitted disease and mucocutaneous disease as previously described [2].

Six possible travel purpose were designed and used in this analysis: tourism; labour, business; research or student; visiting friends and relatives, etc. Travelers were grouped into six geographic areas according to their originating regions: Africa, Asia, Europe, Latin America, North America, and Oceania.

### Statistical analysis

Descriptive statistics were performed for all variables. Continuous variables were summarized as median and range. Proportions were calculated regarding various disease categories, which were compared for the difference among subgroups by Chi-square test. The chisq.test function in the stats package in R was used to implement subgroup comparisons. Incidence rates were calculated as the case number divided by the total number of arriving foreign travelers. We standardized the incidence rate by inbound travel number for each infectious disease, determined the percentage rank, and further represented data as thermodynamic diagrams as previously described [5, 6].

For each reported infectious disease, we aggregated the annual mean case number exported to each province by the source countries/continents, and further constructed origin–destination matrices. The SAS software (version 9.4) was used for data extraction, sorting and cleaning. The R project (version 3.6.3) and relevant packages (such as dplyr, arsenal and ggplot2, etc.) were applied to analyze the data, prepare the tables and draw heatmaps and percentage bar plots [7–9]. ArcGIS software (version 10.5) were used for graphics presentation.

## Results

### Demographic characteristics and infection types of the imported diseases

During 2014–2018, a total of 17,189 travelers with 58 imported infectious diseases were confirmed out of totally 140,210.30 thousand person-times foreign travelers, with an overall incidence of 122.59 per million, which comprised of 14,452 cases reported from CISS and 2,737 reported from NNIDRS (Figure S2). Travelers of male gender, aged 15–44 years, those originating from Asian countries have accounted for a higher proportion of travel related cases than their counterparts (all *p* < 0.001) (Table 1).

**Table 1:** The characteristics of imported cases among foreign travelers in Chinese mainland, 2014‒2018

	**Overall (*n* = 17,189)**	**Respiratory (*n* = 7351)**	**Gastrointestinal (*n* = 282)**	**VBD (*n* = 3128)**	**BSTI (*n* = 6114)**	**Mucocutaneous (*n* = 314)**	***p* value**
**Sex**							< 0.001^a^
Male	12,347 (71.8)	5769 (78.5)	165 (58.5)	1861 (59.5)	4310 (70.5)	242 (77.1)	
Female	4842 (28.2)	1582 (21.5)	117 (41.5)	1267 (40.5)	1804 (29.5)	72 (22.9)	
**Age**	33 (25–45)	35 (25–48)	31 (23–51)	29 (20–41)	33 (26–42)	34 (25–46)	< 0.001^a^
0–14	1485 (8.6)	932 (12.7)	30 (10.6)	469 (15.0)	18 (0.3)	36 (11.5)	
15–29	5326 (31.0)	1755 (23.9)	97 (34.4)	1130 (36.1)	2258 (36.9)	86 (27.4)	
30–44	6040 (35.1)	2425 (33.0)	68 (24.1)	903 (28.9)	2540 (41.5)	104 (33.1)	
45–59	3172 (18.5)	1558 (21.2)	33 (11.7)	472 (15.1)	1050 (17.2)	59 (18.8)	
≥ 60	1166 (6.8)	681 (9.3)	54 (19.1)	154 (4.9)	248 (4.1)	29 (9.2)	
**Destination**							< 0.001^a^
Africa	907 (5.3)	137 (1.9)	8 (2.8)	479 (15.3)	282 (4.6)	1 (0.3)	
Asia	10,105 (58.8)	4833 (65.7)	188 (66.7)	2545 (81.4)	2278 (37.3)	261 (83.1)	
Europe	1342 (7.8)	694 (9.4)	21 (7.4)	22 (0.7)	594 (9.7)	11 (3.5)	
Latin America & Caribbean	165 (1.0)	94 (1.3)	4 (1.4)	19 (0.6)	42 (0.7)	6 (1.9)	
North America	722 (4.2)	520 (7.1)	34 (12.1)	3 (0.1)	157 (2.6)	8 (2.5)	
Oceania	536 (3.1)	468 (6.4)	19 (6.7)	10 (0.3)	26 (0.4)	13 (4.1)	
unknown	3412 (19.8)	605 (8.2)	8 (2.8)	50 (1.6)	2735 (44.7)	14 (4.5)	
**Infection year**							< 0.001^a^
2014	1280 (7.4)	445 (6.1)	10 (3.5)	334 (10.7)	477 (7.8)	14 (4.5)	
2015	2413 (14.0)	1025 (13.9)	16 (5.7)	644 (20.6)	693 (11.3)	35 (11.1)	
2016	3545 (20.6)	1740 (23.7)	63 (22.3)	494 (15.8)	1177 (19.3)	71 (22.6)	
2017	5421 (31.5)	2351 (32.0)	69 (24.5)	1254 (40.1)	1625 (26.6)	122 (38.9)	
2018	4530 (26.4)	1790 (24.4)	124 (44.0)	402 (12.9)	2142 (35.0)	72 (22.9)	

*VBD* Vector-borne disease, *BSTI* Blood/sex- transmitted infection

^a^Test for the categorical variable by Chi-square test

**Fig. 1:**
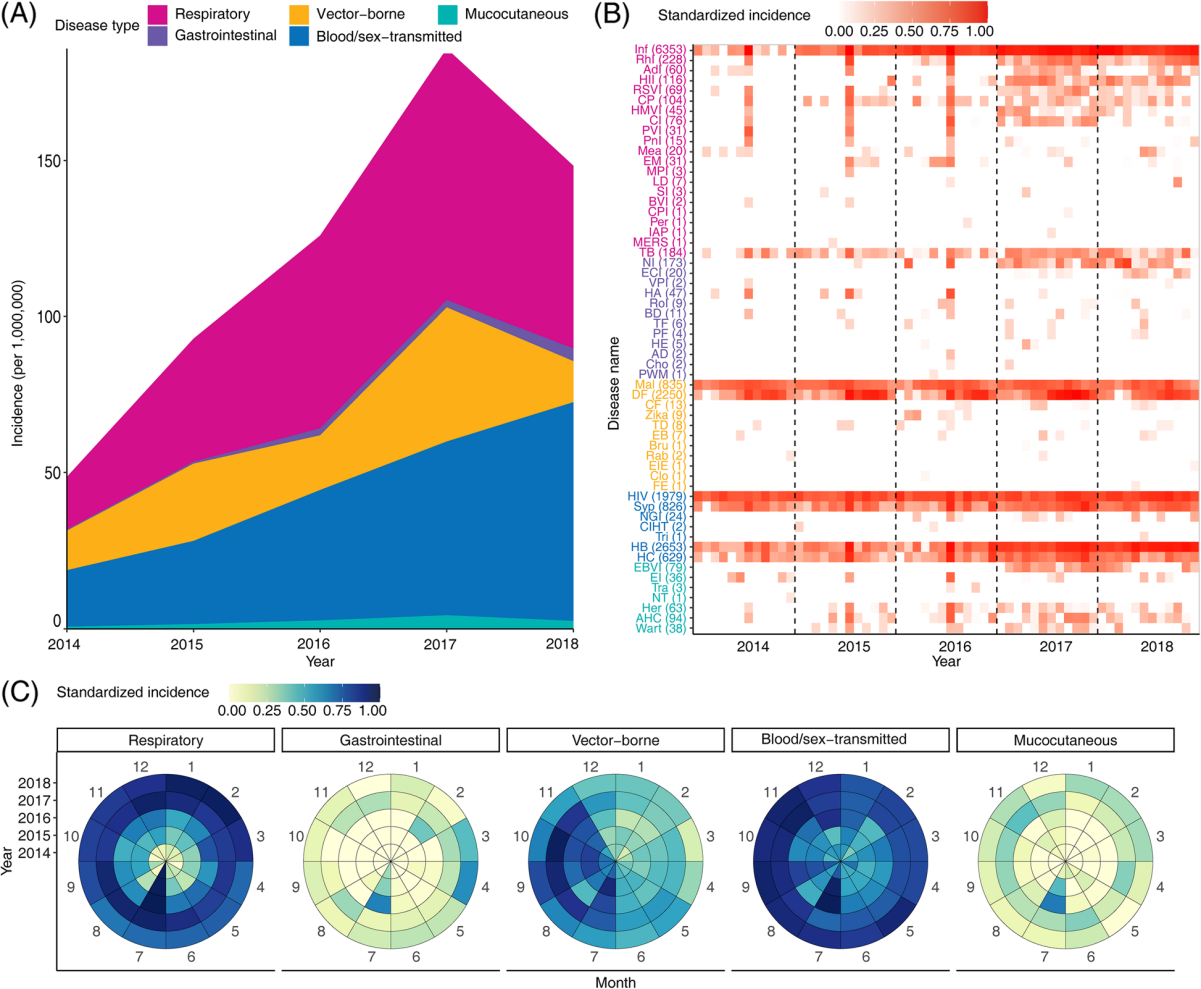
Annual trends and seasonal pattern of imported infections among foreign travelers in the mainland of China, 2014‒2018. **A** Changes in the annual incidence rate of five infection types over time. **B** Changes in the monthly standardized incidence rate of 58 imported infectious diseases over time. **C** Changes in the monthly standardized incidence rate of five infection types over time

The respiratory infection (RI) had the highest average incidence of 24.96 per million and showed ascending tendency over the 5 study years (Fig. 1A). Influenza was most frequently seen among all types of respiratory diseases determined from the travelers, with an extraordinarily high level in the year 2017 and 2018 (Fig. 1B). Blood/sex-transmitted infection (BSTI) was the second most frequent disease with a consistently increasing incidence from 18.10 per million in 2014 to 70.13 per million in 2018. HIV infection, syphilis, hepatitis B and hepatitis C were among the most frequent diagnosis, with incidence rates of 14.12, 5.89, 18.92 and 4.48 per million, respectively (Fig. 1B). When data from two reporting systems were separately analyzed, respiratory infection and vector-borne diseases were the most common syndrome classification from CISS and NNIDRS, respectively (Figure S5).

### The spectrum of imported infection

**Fig. 2:**
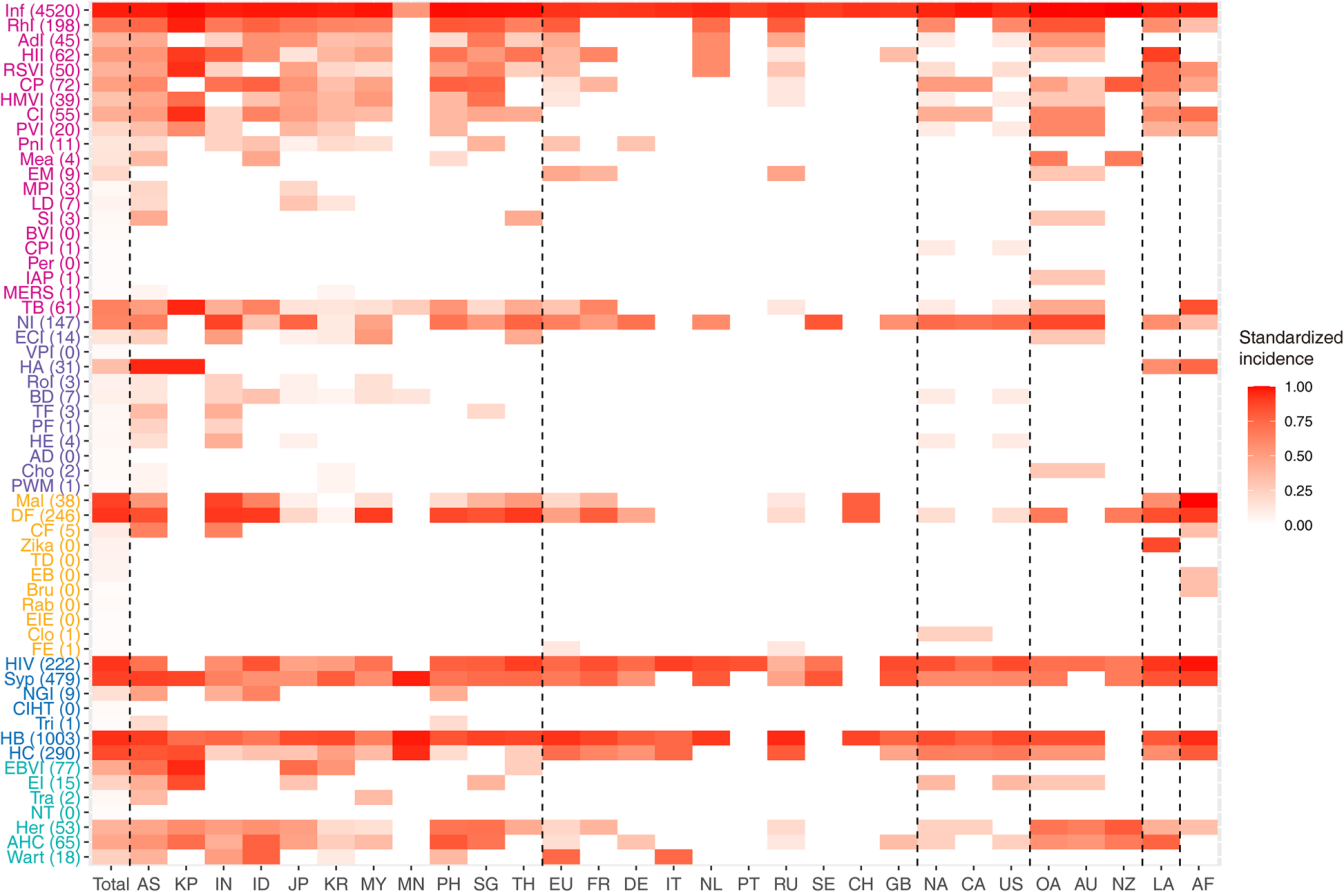
The standardized incidence rate of imported infection from foreign travelers with foreign nationality in 6 continents and 23 selected countries in China. Notes: Inf: Influenza; RhI: Rhinovirus infection; AdI: Adenovirus infection; HII: Haemophilus influenzae infection; RSVI: Respiratory syncytial virus infection; CP: Chicken pox; HMVI: Human metapneumonia virus infection; CI: Coronavirus infections; PVI: Parainfluenza virus infection; PnI: Pneumococcal infection; Mea: Measles; EM: Epidemic mumps; MPI: Mycoplasma pneumoniae infection; LD: Legionella disease; SI: Streptococcal infection; BVI: Boka virus infection; CPI: Chlamydia pneumonia infection; Per: Pertussis; IAP: Infectious atypical pneumonia; MERS: Middle East Respiratory Syndrome; TB: Tuberculosis; NI: Norovirus infection; ECI: Escherichia coli infection; VPI: Vibrio parahaemolyticus infection; HA: Hepatitis A; RoI: Rotavirus infection; BD: Bacterial dysentery; TF: Typhoid fever; PF: Paratyphoid fever; HE: Hepatitis E; AD: Amiba's dysentery; Cho: Cholera; PWM: Pin worm disease; Mal: Malaria; DF: Dengue fever; CF: Chikungunya fever; TD: Tsutsugamushi disease; EB: Encephalitis B; Bru: Brucellosis; Rab: Rabies; EIE: Epidemic and endemic typhus; Clo: Clonorchiasis; FE: Forest encephalitis; Syp: Syphilis; NGI: Neisseria gonorrhoeae infection; CIHT: Chlamydia infection of genitourinary tract; Tri: Trichomoniasis; HB: Hepatitis B; HC: Hepatitis C; EBVI: EB virus infection; EI: Enterovirus infection; Tra: Trachoma; NT: Neonatal tetanus; Her: Herpes; AHC: Acute hemorrhagic conjunctivitis; AS: Asia; KP: Dem. Rep. Korea; IN: India; ID: Indonesia; JP: Japan; KR: Korea; MY: Malaysia; MN: Mongolia; PH: Philippines; SG: Singapore; TH: Thailand; EU: Europe; FR: France; DE: Germany; IT: Italy; NL: Netherlands; PT: Portugal; RU: Russia; SE: Sweden; CH: Switzerland; GB: United Kingdom; NA: North America; CA: Canada; US: United States; OA: Oceania; AU: Australia; NZ: New Zealand; LA: Latin America; AF: Africa

**Fig. 3:**
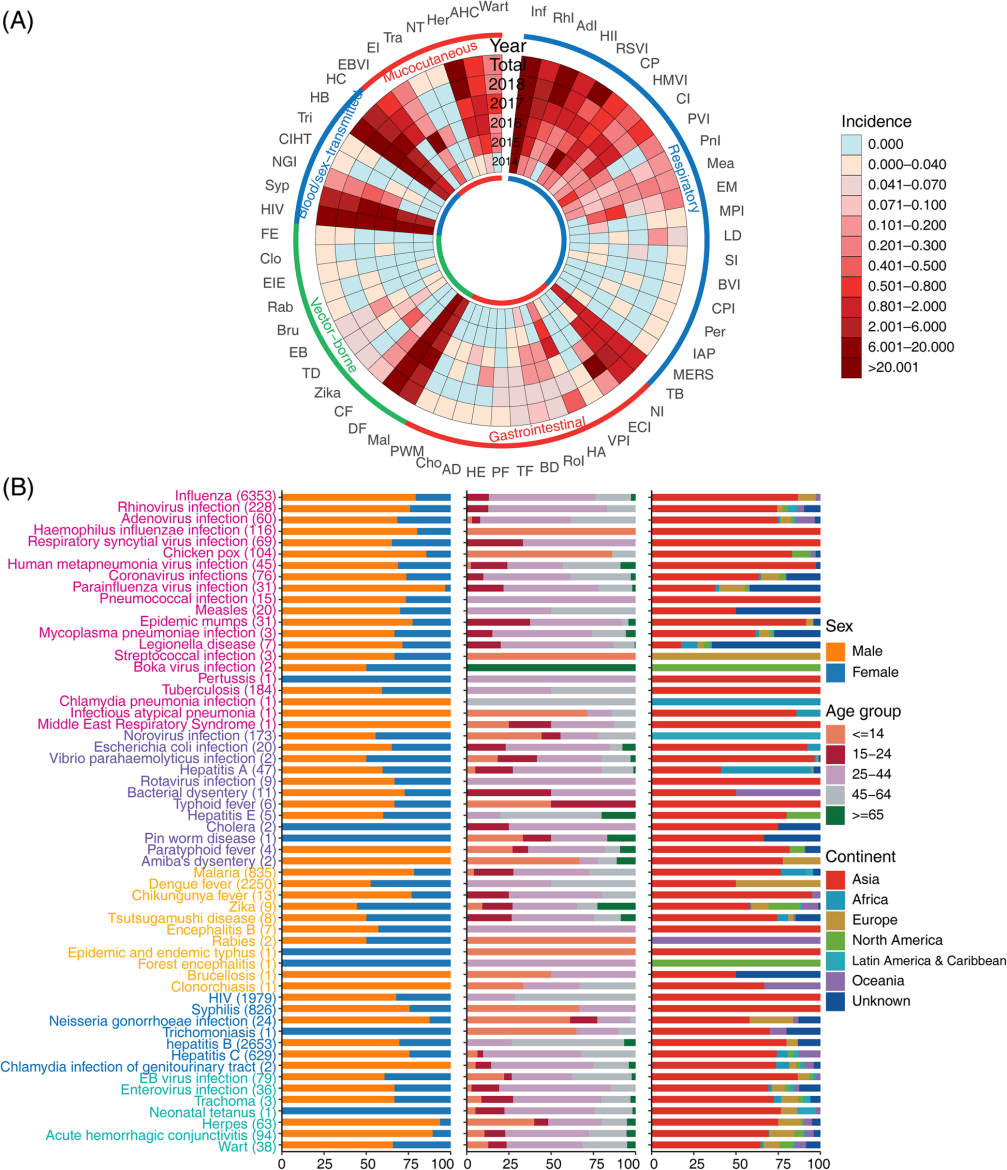
The spectrum of imported infections in foreign travelers with foreign nationality in the mainland of China, 2014–2018. **A** The annual incidence rate of 58 imported infectious diseases. **B** Sex, age, and original continent distribution of 58 different imported infections. Notes: Inf: Influenza; RhI: Rhinovirus infection; AdI: Adenovirus infection; HII: Haemophilus influenzae infection; RSVI: Respiratory syncytial virus infection; CP: Chicken pox; HMVI: Human metapneumonia virus infection; CI: Coronavirus infections; PVI: Parainfluenza virus infection; PnI: Pneumococcal infection; Mea: Measles; EM: Epidemic mumps; MPI: Mycoplasma pneumoniae infection; LD: Legionella disease; SI: Streptococcal infection; BVI: Boka virus infection; CPI: Chlamydia pneumonia infection; Per: Pertussis; IAP: Infectious atypical pneumonia; MERS: Middle East Respiratory Syndrome; TB: Tuberculosis; NI: Norovirus infection; ECI: Escherichia coli infection; VPI: Vibrio parahaemolyticus infection; HA: Hepatitis A; RoI: Rotavirus infection; BD: Bacterial dysentery; TF: Typhoid fever; PF: Paratyphoid fever; HE: Hepatitis E; AD: Amiba's dysentery; Cho: Cholera; PWM: Pin worm disease; Mal: Malaria; DF: Dengue fever; CF: Chikungunya fever; TD: Tsutsugamushi disease; EB: Encephalitis B; Bru: Brucellosis; Rab: Rabies; EIE: Epidemic and endemic typhus; Clo: Clonorchiasis; FE: Forest encephalitis; Syp: Syphilis; NGI: Neisseria gonorrhoeae infection; CIHT: Chlamydia infection of genitourinary tract; Tri: Trichomoniasis; HB: Hepatitis B; HC: Hepatitis C; EBVI: EB virus infection; EI: Enterovirus infection; Tra: Trachoma; NT: Neonatal tetanus; Her: Herpes; AHC: Acute hemorrhagic conjunctivitis

Totally 58 imported infectious diseases were diagnosed among 17,189 travelers (Fig. 2). The 10 most common infections were influenza (with a proportion of 36.96%), followed by hepatitis B (15.43%), dengue fever (13.09%), HIV (11.51%), malaria (4.86%), syphilis (4.81%), hepatitis C (3.66%), rhinovirus infection (1.33%), and tuberculosis (1.07%), and norovirus infection (1.01%), which collectively had taken 93.72% (16,110/17,189) of the confirmed diagnosis. Their annual incidence kept increasing during the study period, especially for HIV infection, hepatitis B and norovirus infection (Fig. 3A).

### The epidemiological features of imported infections

The spectrum of illnesses varied according to age, travel purpose and source country of these travelers. Respiratory illnesses and gastrointestinal illnesses were more likely to be reported in tourism travelers, vector-borne diseases were more likely to occur in laboring travelers, while mucocutaneous (particularly acute hemorrhagic conjunctivitis and EB virus infection) diseases were more likely to occur in travelers with business purposes (Table S1). There was no gender difference for the infection spectrum.

By pooling the five-year data, an obvious seasonal pattern was shown that the monthly standardized incidence of all 5 types of infection peaked in July (Fig. 1C), and respiratory disease had the highest average incidence rate (245.00 per million) in this month, followed by blood/sex-transmitted disease (95.80 per million), vector-borne disease (40.58 per million), mucocutaneous disease (7.54 per million), gastrointestinal disease (6.67 per million). This trend was remarkably observed for gastrointestinal disease, mucocutaneous disease and vector-borne disease with remarkable monthly difference than other diseases (Fig. 1C).

### The source countries and inbound provinces of imported travelers

**Fig. 4:**
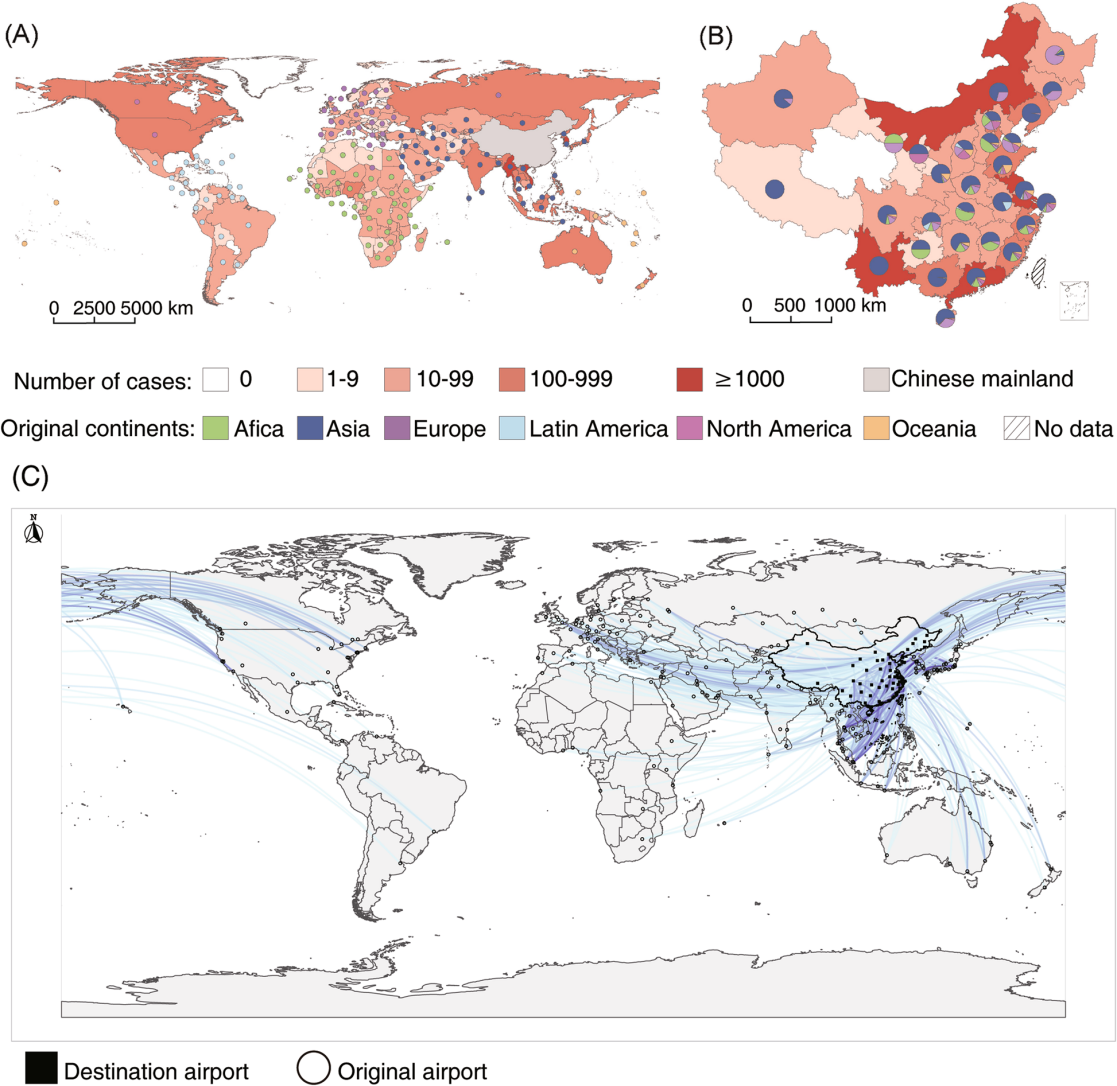
The originating countries and destiny provinces in China of foreign travelers with imported infections. **A** Distribution of source countries of imported infections. **B** The distribution of imported infections in each province of China and the proportion of their original continent. **C** The distribution map of major international flights and passenger flow entering China from foreign countries or regions

Altogether 17,120 ill travelers had reported their source countries, which were originated from 152 countries on six continents, with their destination distributed in all 31 provinces in the mainland of China (Fig. 4A and B). Asian travelers had taken the predominant part, accounting for 59.02% (10,105/17120) of all imported cases, followed by those from European and African countries. Within Asian countries, Korean travelers had contributed the highest number of cases with RIs, BSTIs and VBDs. The destination provinces which had reported more than 1000 imported cases included Yunnan (3,134 cases), Guangdong (2,017 cases), Shanghai (1,939 cases), Inner Mongolia (1,447 cases) and Jiangsu (1,348 cases).

The major international flights and traveler inflow from foreign countries or regions were mapped (Fig. 4C). In 2016, the annual traveler flow from foreign international airports to Chinese airports was about 77.09 million. Among them, the continent with the largest annual inflow number of international flights to China is Asia (62.80 million), followed by North America (5.62 million), Europe (5.60 million), Oceania (1.80 million), Africa (1.00 million), and Latin America (0.27 million). The movement of imported infection followed specific routes (Fig. 4), in that the connections were more frequently seen between African and Hubei, Guizhou and Jiangxi province (13 cases on average per year), and between Asian (mainly including Burma, Vietnam and Laos) and Yunnan province (592 cases on average per year,) and between Asian (Mongolia and Russia) and Inner Mongolia (233 cases on average per year). Notably, the imported patients travelling to metropolises such as Beijing, Shanghai and Guangdong Provinces tended to be multi-continental sources (Fig. 4B and C). The annual travelling data were available from six continents and 23 countries, which were used as denominator for estimation of incidence. This had accounted for 79.25% (111.12/140.21 million person-times) of total travelling population and 45.96% (7,900/17,189) of total imported cases. When related to the source regions, the highest case incidence was reported from the African travelers (296.00 per million), in contrast with the highest case number from Asia, while the lowest incidence was reported from European countries (41.70 per million). When specific diagnosis was compared, both the absolute case number and incidence rate was observed for influenza (Fig. 2).

## Discussion

Globalization has promoted the growth of population movements between countries, which has changed the global disease spectrum and brought a huge impact on the public health. In China, imported infectious diseases remain the major cause of morbidity and mortality, partially owing to the close connection of China with the rest of the world through travel and trade. The previous publications that were built on the real-time monitoring system from the enter-exit port have provided a snapshot of travel related infection over a geographic area, or time frame, however, not differentiate between native citizens and foreigner travelers for their role in spreading the imported infectious diseases.

In this study, rather than study all international travelers, we focused on the foreign travelers for analysis, comparing the imported diseases by their travel related data to identify countries, seasons and travel modes that were associated with a high incidence of imported infections. We determined that Asian travelers contributed the highest number of incoming travelers and cases with imported infectious diseases probably due to the geographical proximity and frequent international exchanges. In contrast, African travelers had the highest incidence of imported infection, thus posing as the high priority in inbounding quarantine. The imported patients were primarily originated from bordering countries of China, with a high possibility of causing local epidemics [10], such as importation of dengue from Vietnam, Laos and Myanmar into Yunnan, and blood/sex-transmitted diseases from Mongolia and Russia into Inner Mongolia, respectively. In contrast, the imported patients reported in metropolises or provinces such as Beijing, Shanghai and Guangdong Provinces tended to from a wide range of source countries, due to a highly complicated transportation exchange around the world.

The spectrum of imported infectious diseases from foreign travelers also varied depending on the travel season and the travel purposes. Except for vector-borne diseases that were extraordinarily overrepresented between July and November, all other infectious diseases were evenly detected throughout the year, posing a persistent burden to the medical quarantine work. The major epidemic peak of VBD in travelers might reflect the epidemic pattern of the disease in the original endemic areas. For example, the peaking season of VBD in southeast Asia spanned from June to September, which coincided with those of the imported VBD [11, 12]. In the case of most important VBDs, dengue disease generally peaked in June and September in Southeast Asian countries [11, 12] while malaria cases generally peaked in September to November in African countries [13], which might be responsible for the high and sustained epidemic peak of imported VBDs from July to November. Previous reports on the imported malaria in the UK [14], Finland [15] and Bulgaria [16], and the imported dengue in Japan [17], Spain [18] and East London [19], had revealed similar epidemic patterns as in the current study. Travelers to dengue-/malaria-endemic countries appear to serve as reliable “sentinels” that may inform the international community of the onset of epidemic activity in specific areas.

During the study period, the incidence of imported infections from foreign travelers had been increasing, especially for gastrointestinal and blood/sex-transmitted diseases. In agreement with the previous report that analyzed all the native and foreign cases [2], respiratory infections were the most frequent diagnosis among five infection types. In addition to the most common diseases including influenza and rhinovirus infections, the diseases with a small number of imported cases but with epidemic potential and risk of high mortality among humans should be given prioritized attention, such as MERS [20]. Notably, the majority of VBDs were identified by the NNIDS instead of through the custom screening, likely due to the relatively long incubation period of most VBD, which was consistent with a previous study [3], indicating the necessity to inquiry recent travel history of suspected foreign cases after their international travel that is helpful for timely and accurate diagnosis, treatment and management of them. Although malaria is no longer endemic in China, the large number and continuous import of malaria have brought great challenges to the goal of a national malaria elimination by 2020 [21]. Emerging imported diseases in recent years such as zika and chikungunya also posed serious threats to human health, therefore enhanced screening among foreign travelers should be stressed in the high risky population and seasons as we have determined in the current research. In addition, specific disease surveillance and pathogen detection need to be strengthened according to the origin of foreign travelers. For example, it is necessary to strengthen the surveillance of vector-borne pathogens among passengers from Africa, as well as influenza and other respiratory diseases among passengers from European and American countries.

Our study was subject to several limitations. First, the incidence rate might be underreported for most imported infectious diseases because many patients suffering from post-travel symptoms might be missed from the inbound screening, and not seek medical care due to mild symptoms thereafter, or even if they go to the doctor, they were not judged as travel-related illnesses. Second, we could not calculate the incidence rate of foreign travelers regarding their age, sex or original country since the detailed denominator data were inaccessible. Third, the specific location of the infection was difficult to determine for those travelers who have visited several countries before entering China (We used the most recent country they visited as the original country). For example, the imported dengue or malaria cases from France, Germany, or Switzerland, where no endemic diseases exist, were quite suspicious.

## Conclusions

In conclusion, our study gave a profile of imported infectious diseases among foreign travelers to China, and pinpointed the target regions, seasons, populations for imported disease prevention and control, which might assist in making medical risk assessments and identifying imported cases, especially those of highly communicable and highly lethal infections. The findings from the current study might also shed light on the prevention and control of imported infection in other countries in the world.

## Supplementary Information

**Additional file 1: Figure S1.** The distribution of all 272 entry-exit ports in Chinese mainland. **Figure S2.** The flow chart on the data extraction process and criteria of imported infections in mainland of China, 2014–2018. **Figure S3.** The classification of infectious diseases at ports of Chinese mainland. **Figure S4.** Report card of infectious diseases of the People’s Republic of China. **Figure S5.** The disease spectrum of during-travel infectious diseases (A) and post-travel infectious diseases (B) in relation with the inbound provinces in Chinese mainland China, 2014–2018. **Table S1.** The travel reason for during-travel cases of five type of diseases in Chinese mainland, 2014–2018.

## Data Availability

Raw data are not publicly available and are protected due to data privacy laws, which were used under license for the current study, but are available upon reasonable request to the corresponding author. The request will be responded within one week.

## Abbreviations

HIV: Human Immunodeficiency Virus
TB: Tuberculosis
MERS: Middle East Respiratory Syndrome
ITHC: International Travel Healthcare Center
NNIDRS: National Notifiable Infectious Disease Reporting System
CCDC: Chinese Center for Diseases Control and Prevention
COVID-19: Coronavirus disease 2019

## References

[1] Tatem AJ, Jia P, Ordanovich D, Falkner M, Huang Z, Howes R (2017). The geography of imported malaria to non-endemic countries: a meta-analysis of nationally reported statistics. Lancet Infect Dis.

[2] Fang LQ, Sun Y, Zhao GP, Liu LJ, Jiang ZJ, Fan ZW (2018). Travel-related infections in mainland China, 2014–16: an active surveillance study. The Lancet Public health.

[3] Wang Y, Wang X, Liu X, Ren R, Zhou L, Li C (2018). Epidemiology of Imported Infectious Diseases, China, 2005–2016. Emerg Infect Dis.

[4] Wu Y, Liu MY, Wang JL, Zhang HY, Sun Y, Yuan Y (2020). Epidemiology of imported infectious diseases, China, 2014–18. Journal of travel medicine..

[5] Yang S, Wu J, Ding C, Cui Y, Zhou Y, Li Y (2017). Epidemiological features of and changes in incidence of infectious diseases in China in the first decade after the SARS outbreak: an observational trend study. Lancet Infect Dis.

[6] Yu J, Lai S, Geng Q, Ye C, Zhang Z, Zheng Y (2019). Prevalence of rotavirus and rapid changes in circulating rotavirus strains among children with acute diarrhea in China, 2009–2015. J Infect.

[7] Wickham H (2016). ggplot2: Elegant graphics for data analysis.

[8] Heinzen E, Sinnwell J, Atkinson E, Gunderson T, Dougherty G. arsenal: An Arsenal of 'R' Functions for Large-Scale Statistical Summaries; 2021: https://cran.r-project.org/web/packages/arsenal/index.html.Available from

[9] Wickham H, François R, Henry L, Müller K. dplyr: A Grammar of Data Manipulation; 2022: https://CRAN.R-project.org/package=dplyr.Available from

[10] Hu TS, Zhang HL, Feng Y, Fan JH, Tang T, Liu YH (2017). Epidemiological and molecular characteristics of emergent dengue virus in Yunnan Province near the China-Myanmar-Laos border, 2013–2015. BMC Infect Dis.

[11] Phanitchat T, Zhao B, Haque U, Pientong C, Ekalaksananan T, Aromseree S (2019). Spatial and temporal patterns of dengue incidence in northeastern Thailand 2006–2016. BMC Infect Dis.

[12] Cuong HQ, Vu NT, Cazelles B, Boni MF, Thai KT, Rabaa MA (2013). Spatiotemporal dynamics of dengue epidemics, southern Vietnam. Emerg Infect Dis.

[13] Diouf I, Rodriguez Fonseca B, Caminade C, Thiaw WM, Deme A, Morse AP (2020). Climate Variability and Malaria over West Africa. Am J Trop Med Hyg.

[14] Nabarro LEB, Nolder D, Broderick C, Nadjm B, Smith V, Blaze M (2018). Geographical and temporal trends and seasonal relapse in Plasmodium ovale spp. and Plasmodium malariae infections imported to the UK between 1987 and 2015. BMC medicine..

[15] Guedes S, Siikamäki H, Kantele A, Lyytikäinen O (2010). Imported malaria in Finland 1995 to 2008: an overview of surveillance, travel trends, and antimalarial drug sales. J Travel Med.

[16] Rainova IG, Harizanov RN, Kaftandjiev IT, Mikov OD, Tsvetkova ND (2018). Imported malaria in Bulgaria, status and prognosis after eradication in 1965. J Infect Public Health.

[17] Fukusumi M, Arashiro T, Arima Y, Matsui T, Shimada T, Kinoshita H (2016). Dengue Sentinel Traveler Surveillance: Monthly and Yearly Notification Trends among Japanese Travelers, 2006–2014. PLoS Negl Trop Dis.

[18] Redondo-Bravo L, Ruiz-Huerta C, Gomez-Barroso D, Sierra-Moros MJ, Benito A, Herrador Z (2019). Imported dengue in Spain: a nationwide analysis with predictive time series analyses. Journal of travel medicine..

[19] Riddell A, Babiker ZO (2017). Imported dengue fever in East London: a 6-year retrospective observational study. Journal of travel medicine..

[20] Wu J, Yi L, Zou L, Zhong H, Liang L, Song T, et al. Imported case of MERS-CoV infection identified in China, May 2015: detection and lesson learned. Euro surveillance : bulletin Europeen sur les maladies transmissibles = European communicable disease bulletin. 2015;20(24):21158. 10.2807/1560-7917.es2015.20.24.21158 26111235

[21] China NHCo. Beijing: Action plan of China malaria elimination (2010–2020). 2015 http://www.nhcgovcn/jkj/s5873/201005/f84f1c4b0f32420990d23b65a88e2d87.shtml.

