# Supplementary material for: The imported infections among foreign travelers in China: an observational study
Source: Global Health. 2022 Nov 24;18:97. doi: 10.1186/s12992-022-00893-7 (PMC9701002; doi:10.1186/s12992-022-00893-7)
Supplement: Supplementary file 1 — Additional file 1: Figure S1. The distribution of all 272 entry-exit ports in Chinese mainland. Figure S2. The flow chart on the data extraction process and criteria of imported infections in mainland of China, 2014–2018. Figure S3. The classification of infectious diseases at ports of Chinese mainland. Figure S4. Report card of infectious diseases of the People’s Republic of China. Figure S5. The disease spectrum of during-travel infectious diseases (A) and post-travel infectious diseases (B) in relation with the inbound provinces in Chinese mainland China, 2014–2018. Table S1. The travel reason for during-travel cases of five type of diseases in Chinese mainland, 2014–2018. [file 12992_2022_893_MOESM1_ESM.docx]

**Figure S1. The distribution of all 272 entry-exit ports in Chinese mainland.**

The red, brown and purple points indicate the airports, water ports and land entry-exit stations, respectively.


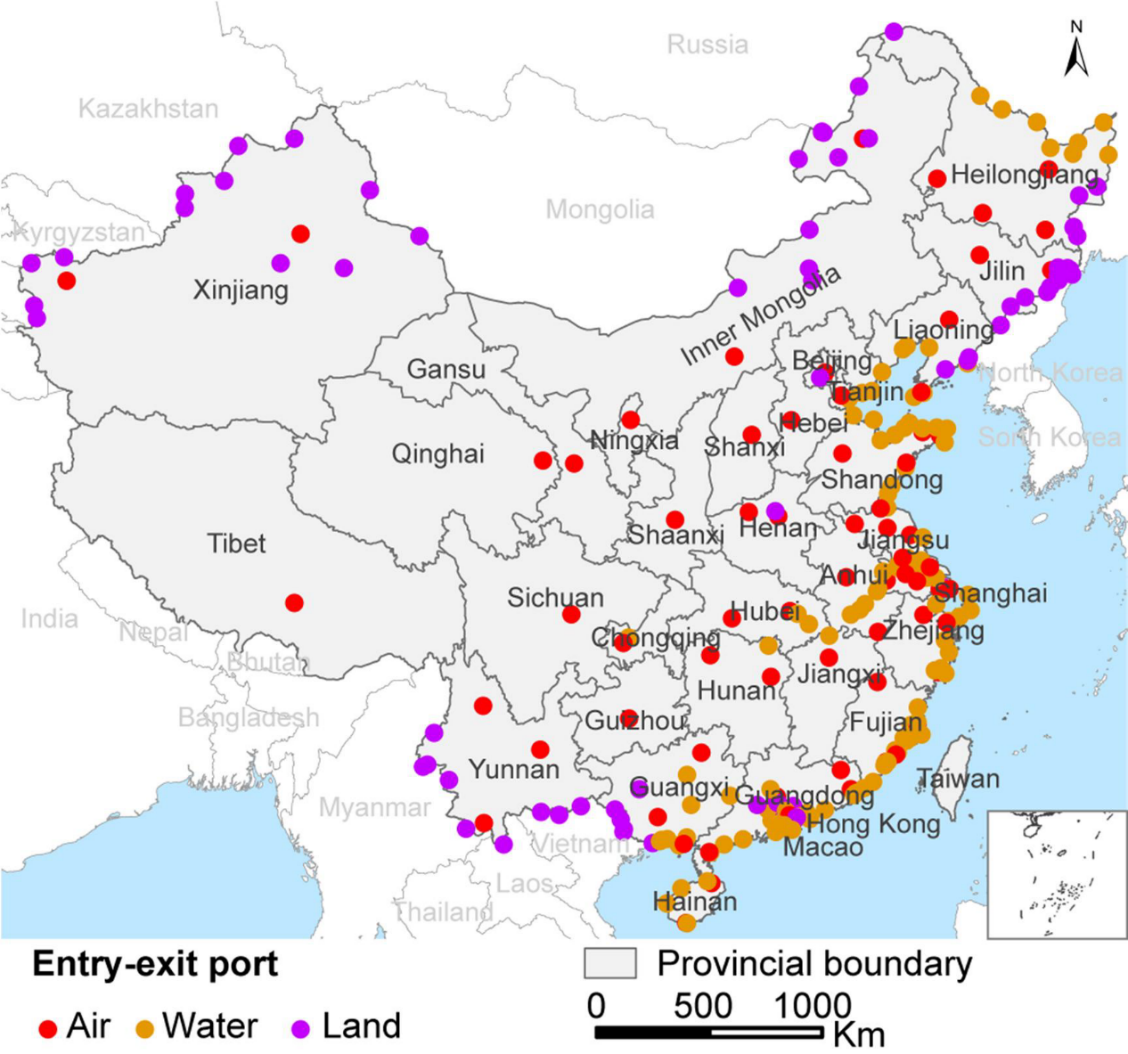


**Figure S2. The flow chart on the data extraction process and criteria of imported infections in mainland of China, 2014–2018**

**Figure S3. The classification of infectious diseases at ports of Chinese mainland.**

All imported infection

Excluding the possibility of infectious diseases

Infectious diseases suspected of being transmitted by other routes

Symptoms: fever, muscle pain, joint pain, headache, petechiae, ecchymosis, rash, jaundice;

Signs: three red signs;

Combined with epidemiological investigations

Symptoms: fever, nausea, vomiting, abdominal pain, diarrhea, bloody stool;

Signs: skin elasticity test, dry lips;

Combined with epidemiological investigations

Symptoms: fever, severe headache, jet vomiting;

Signs: neck ankylosis, Kernig's sign positive, Brudzinski's sign positive;

Combined with epidemiological investigations

Symptoms: fever, cough, chest pain, expectoration, hemoptysis, shortness of breath, dyspnea;

Signs: dry and wet lungs;

Combined with epidemiological

investigations

Further investigation and disposal in accordance with the “Technical Plan for the Investigation and Disposal of **Gastrointestinal Infectious Diseases** at Ports”

Further investigation and disposal in accordance with the “Technical Plan for the Investigation and Disposal of **Vector-borne Infectious Diseases** at Ports”

**Further investigation and disposal** in accordance with relevant regulations

Register information, give health advice, issue a "convenient card for health care", **release**

Further investigation and disposal in accordance with the “Technical Plan for the Investigation and Disposal of **Respiratory Infectious Diseases** at Ports”

Follow-up investigation

File archiving

**Figure S4. Report card of infectious diseases of the People’s Republic of China.**


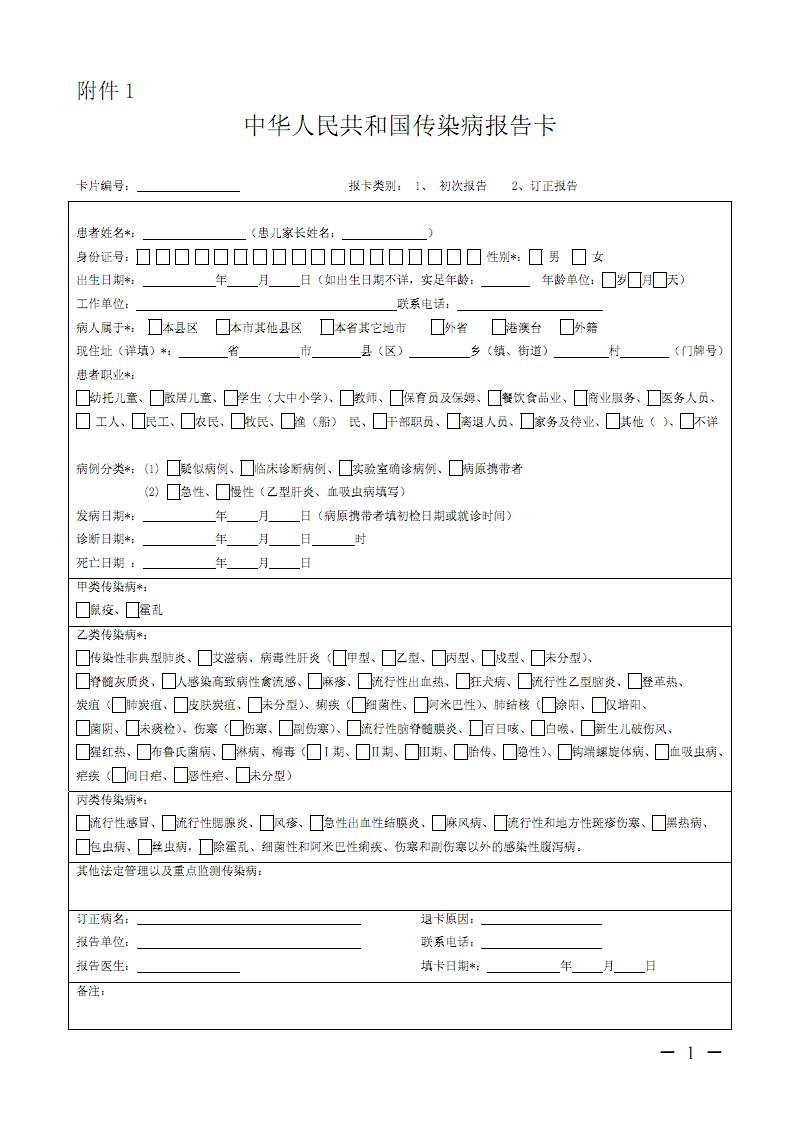


**Figure S5. The disease spectrum of during-travel infectious diseases (A) and post-travel infectious diseases (B) in relation with the inbound provinces in Chinese mainland China, 2014–2018.**


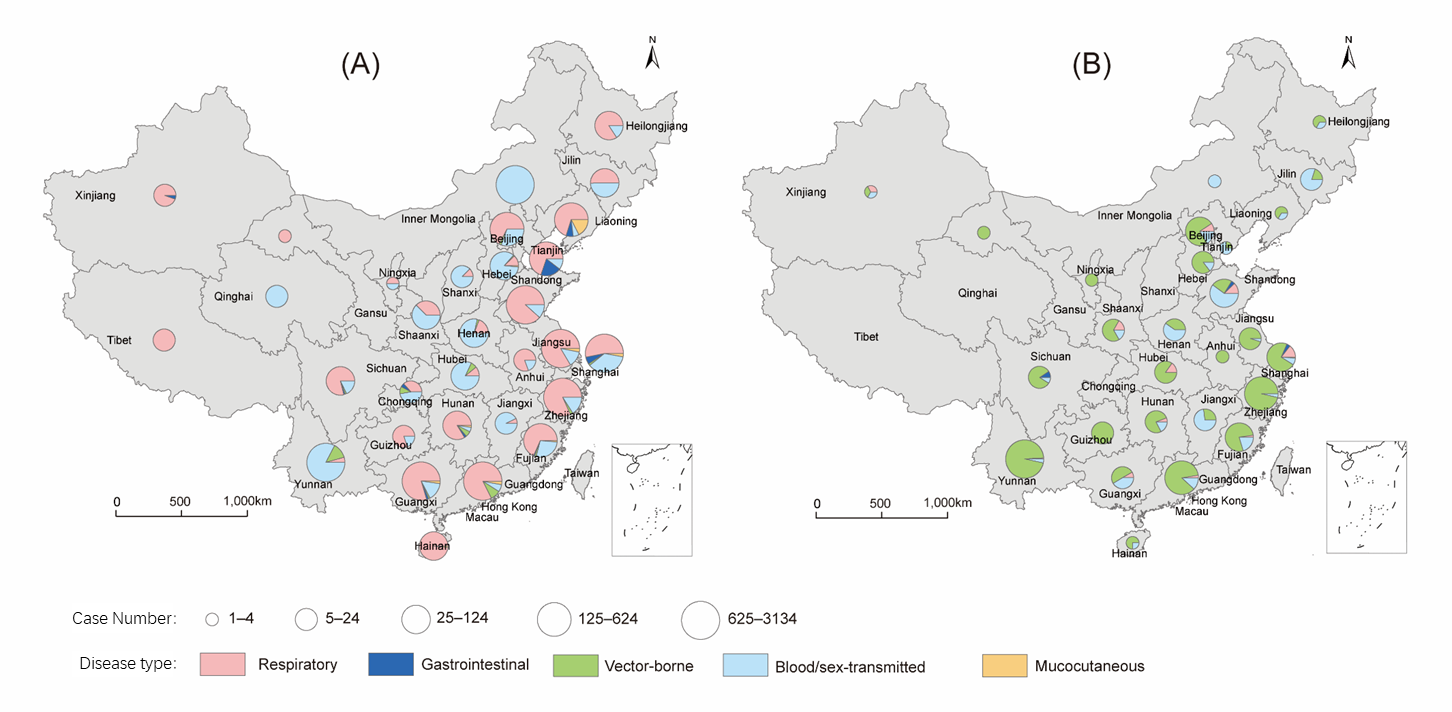


**Table S1. The travel reason for during-travel cases of five type of diseases in Chinese mainland, 2014–2018**

|  | **Total (N=14452)** | **Respiratory (N=7294)** | **Gastrointestinal (N=267)** | **Vector-borne (N=670)** | **Blood/sex-transmitted (N=5921)** | **Mucocutaneous (N=300)** | **p value** |
| --- | --- | --- | --- | --- | --- | --- | --- |
| **Trip reason** |  |  |  |  |  |  | < 0.001 |
| Tourism | 1699 (11.8) | 1309 (17.9) | 77 (28.8) | 68 (10.1) | 226 (3.8) | 19 (6.3) |  |
| Labour | 1556 (10.8) | 791 (10.8) | 36 (13.5) | 173 (25.8) | 517 (8.7) | 39 (13.0) |  |
| Sailor | 1085 (7.5) | 993 (13.6) | 3 (1.1) | 14 (2.1) | 37 (0.6) | 38 (12.7) |  |
| Business | 1053 (7.3) | 465 (6.4) | 33 (12.4) | 52 (7.8) | 453 (7.7) | 50 (16.7) |  |
| Visiting friends or relatives | 406 (2.8) | 316 (4.3) | 5 (1.9) | 56 (8.4) | 17 (0.3) | 12 (4.0) |  |
| Research or student | 187 (1.3) | 137 (1.9) | 6 (2.2) | 19 (2.8) | 24 (0.4) | 1 (0.3) |  |
| Others^*^ | 1662 (11.5) | 886 (12.1) | 34 (12.7) | 136 (20.3) | 556 (9.4) | 50 (16.7) |  |
| Missing | 6804 (47.1) | 2397 (32.9) | 73 (27.3) | 152 (22.7) | 4091 (69.1) | 91 (30.3) |  |

^*^Others indicate missionary, volunteer, aid work, and immigration.
